# Purification and characterization of an extracellular β‐glucosidase from *Sporothrix schenckii*

**DOI:** 10.1002/2211-5463.12108

**Published:** 2016-10-06

**Authors:** Alicia Hernández‐Guzmán, Alberto Flores‐Martínez, Patricia Ponce‐Noyola, Julio C. Villagómez‐Castro

**Affiliations:** ^1^ Departamento de BiologíaDivisión de Ciencias Naturales y ExactasUniversidad de GuanajuatoMéxico

**Keywords:** biochemical characterization, purification, *Sporothrix schenckii*, β‐glucosidase

## Abstract

An extracellular β‐glucosidase (E.C. 3.2.1.21), induced by cellulose in the mycelial form of human pathogen fungus *Sporothrix schenckii,* was purified to homogeneity using hydroxyapatite (HAp) adsorption chromatography in batch and Sephacryl S200‐HR size exclusion chromatography. The molecular mass of the purified enzyme was estimated to be 197 kDa by size exclusion chromatography with a subunit of 96.8 kDa determined by SDS/PAGE. The β‐glucosidase exhibited optimum catalytic activity at pH 5.5/45 °C and was relatively stable for up to 24 h at 45 °C. Isoelectric focusing displayed an enzyme with a pI value of 4.0. Its activity was inhibited by Fe^2+^ but not by any other ions or chelating agents. *K*_m_ and *V*_max_ values of the purified enzyme were 0.012 mm and 2.56 nmol·min^−1^·mg^−1^, respectively, using 4‐methylumbelliferyl β‐D‐glucopyranoside (4‐MUG) as the substrate and 44.14 mm and 22.49 nmol·min^−1^·mg^−1^ when *p*‐nitrophenyl β‐D‐glucopyranoside (*p*‐NPG) was used. The purified β‐glucosidase was active against cellobioside, laminarin, 4‐MUG, and *p*‐NPG and slightly active against 4‐methylumbelliferyl β‐D‐cellobioside and *p*‐nitrophenyl β‐D‐cellobioside but did not hydrolyze 4‐methylumbelliferyl β‐D‐xyloside, 4‐methylumbelliferyl β‐D‐galactopyranoside nor 4‐methylumbelliferyl α‐D‐glucopyranoside. In addition, the enzyme showed transglycosylation activity when it was incubated along with different oligosaccharides. Whether the transglycosylation and cellulase activities function *in vivo* as a mechanism involved in the degradation of cellulolytic biomass in the saprophytic stage of *S. schenckii* remains to be determined.

**Abbreviations**
4‐MU: 4‐methylumbelliferyl
4‐MUC: 4‐methylumbelliferyl β‐D‐cellobioside
4‐MUG: 4‐methylumbelliferyl β‐D‐glucopyranoside
HAp: hydroxyapatite
*p‐*NPC: *p*‐nitrophenyl β‐D‐cellobioside
*p*‐NPG: *p*‐nitrophenyl β‐D‐glucopyranoside

*Sporothrix schenckii* is a dimorphic fungus within the family Ophiostomataceae able to infect humans and other mammals 1, 2. It is part of a species complex named *Sporothrix schenckii* complex along with *S. globosa, S. brasiliensis, S. schenckii sensu stricto, S. luriei, S. albicans, S. mexicana, S. brunneoviolacea*, and *S. pallida* 3, 4. This fungus is widely distributed in nature and grows as a saprophyte on dead or decomposing matter and also shows association with plants 5, 6. As other filamentous fungi, it is able to degrade biomass and organic matter due to its protein secretion system 7, 8, 9. One of the principal polymers and the main structural component of plant cell wall is the cellulose, a linear polymer of β‐(1,4)‐d‐glucose residues which can be hydrolyzed by different cellulases produced by several microorganisms in order to obtain glucose. One of the enzymes secreted by filamentous fungus 10, 11 and other microorganisms 12, 13 helping to degrade the cellulose is the β‐glucosidase. This enzyme releases glucose by acting on cello‐oligosaccharides originated by the action of other enzymes of the cellulolytic enzyme complex. Up to date, the most studied enzymes on *S. schenckii* are the proteinases. In the yeast parasitic form, it was determined that the proteolytic activity by different species of *Sporothrix* 14 and two proteinases were purified 15. Hydrolytic activity of different enzymes such as acid phosphatase 16, dextranase 17, catalase, urease, and gelatinase 14 in *S. schenckii* has been also detected.

Most of the studies performed on this fungus have been focused on its yeast phase due to its medical importance 1, 18, 19, therefore, its saprophytic stage has not been extensively studied and little is known about its metabolism in their saprophytic stage. In order to get an approach to its physiology and its poorly known saprophytic stage, we report the purification and characterization of the first extracellular enzyme from *S. schenckii* involved in the degradation of cellulolytic biomass.

## Experimental procedures

### Strains and culture conditions

*Sporothrix schenckii,* strain EH‐206 kindly provided by Dra. Conchita Toriello (UNAM, Unidad de Micología, Mexico), was maintained on Yeast extract Peptone Dextrose agar (YPD) at 28 °C. For cellulase production, 1 × 10^6^ conidia per mL of *S. schenckii* were inoculated in Erlenmeyer flasks containing Mathur's medium: 10 mm MgSO_4_·7H_2_O; 20 mm KH_2_PO_4_; 35 mm l‐glutamic acid and 0.2% glucose, supplemented with 1% or 2% cellulose (Sigmacell, type 101; Sigma, St. Louis, MO, USA); final pH 4.5, and incubated at 28 °C with continuous shaking (120 r.p.m.). For enzymatic activity induction, we used a 125‐mL Erlenmeyer flask containing 40 mL of medium and 2‐L Erlenmeyer flasks with 600 mL of medium for the β‐glucosidase purification.

### Sample preparation

For protein quantification and activity test, the samples were prepared as described below. After incubation, cultures were filtered through Whatman # 1 filter paper, the cell‐free growth medium was freeze dried and dissolved in a small volume of deionized water. The samples were centrifuged at 3000 ***g*** for 10 min to remove remaining cellulose. Low molecular weight metabolites and salts were removed by eluting the supernatant through a column (1.0 × 10 cm) of Bio‐Gel P‐10 with 10 mm phosphate buffer, pH 7.0. This sample, filtered and concentrated cell‐free extract, was denominated CFCF, fractions with β‐glucosidase activity were stored at 4 °C until the protein purification and quantification.

### Induction of β‐glucosidase activity

To establish the optimal conditions for cellulolytic activities like induction, we performed growth kinetics and measured the β‐glucosidase activity at different times ranging from 12, 24, 48, 72, and 120 h, using 2% cellulose as the sole carbon source or glucose at the same concentration as a control. Fungal growth was measured as mycelium protein since residual cellulose present in the medium interfered with dry weight quantitation.

### Purification of β‐glucosidase

β‐glucosidase purification was carried out at 4 °C. *Sporothrix schenckii* was grown during a period of 3 days, 3 L of medium was used as the starting material for enzyme purification. The desalted and concentrated sample was applied on 1 mL of hydroxyapatite (HAp) adsorption chromatography in batch, equilibrated with 10 mm phosphate buffer, pH 7, and eluted with a discontinuous phosphate buffer gradient. Fractions with positive activity were freeze dried, resuspended in 1 mL of deionized water, and eluted with 50 mm phosphate buffer, pH 7 in a Sephacryl S200‐HR size exclusion chromatography column (1 × 119 cm, 93.5 mL). Fractions with β‐glucosidase activity were pooled and used for biochemical characterization.

### Enzyme assay

β‐glucosidase activity was measured by a fluorometric method using 4‐MUG (Sigma) as substrate 20. Reaction mixture consisting of 4‐MUG (5 μm), enzyme fraction, and 100 mm citrate buffer, pH 5.0, in a final volume of 200 μL were incubated at 45 °C. After 1 h, the reaction was stopped with 2.5 mL of 0.5 m Na_2_CO_3_ buffer, pH 10.4 and the 4‐methylumbelliferone (4‐MU) released was measured in a Perkin–Elmer LS‐5B luminescence spectrometer with excitation and emission set at 350 and 440 nm, respectively. β‐glucosidase activity was expressed in terms of specific activity as nmol of 4‐MU·min^−1^·mg^−1^ protein. To determine the specificity of the purified enzyme, its activity was tested against *p‐*NP‐glycosides, using *p‐*NPG and *p‐*NPC (Sigma) as substrates 21. Briefly, the enzyme fraction, the substrate at 5 mm final concentration and 100 mm citrate buffer, pH 5.0 in a final volume of 200 μL were mixed and incubated for 1 h at 45 °C. The reaction was stopped with 2.5 mL of 0.5 m Na_2_CO_3_ buffer, pH 10.4 and the *p‐*NP released was measured at 405 nm. The specific activity was expressed as nmol of *p*‐NP liberated per minute per milligram of protein. The purified enzyme was also tested against the natural substrates, cellobiose, and laminarin (Sigma). In this case the activity was assayed using the 3,5‐dinitrosalicylic acid (DNS) method for reducing sugar analysis using glucose as the standard 22. The reaction containing the purified enzyme, 50 μL of 1% substrate in 100 mm citrate buffer, pH 5.0 was carried out at 45 °C for 1 h. 300 μL of DNS reagent was added and the samples were boiled for 10 min. The color developed was measured at 540 nm. The enzyme activity was defined as the nmol of glucose liberated per minute per milligram of protein.

### Protein quantitation

Protein concentration was estimated by the Bicinchoninic acid method 23 and Lowry's method 24 using bovine serum albumin as standard. Protein in the column eluates was monitored by measuring A_280_.

### Determination of molecular mass by gel filtration

The molecular mass of the native protein was estimated by gel filtration on Sephacryl S200‐HR column, calibrated with: thyroglobulin (*M*_r_ 669 000), β‐amylase (*M*_r_ 200 000), ADH (*M*_r_ 150 000), bovine serum albumin (*M*_r_ 66 000), carbonic anhydrase (*M*_r_ 29 000), cytochrome c (*M*_r_ 12 000), and cyanocobalamin (*M*_r_ 1350) as standard proteins (Sigma).

### pI determination

The pI was determined using the Rotofor^®^ System by Bio‐Rad according to the manufacturer instructions using Bio‐Lyte^®^ ampholytes in the pH range 3–10.

### Effects of metal ions and chelating agents on the enzyme activity

Effects of divalent metal ions and chelating agents on the β‐glucosidase activity were also evaluated. These were studied by determining the activity of the purified β‐glucosidase toward 4‐MUG in the presence of cations or chelant agents at different concentration (2.5–10 mm). The experiment was conducted in triplicate.

### Thermostability

The thermostability of the purified enzyme was determined by incubating the enzyme in 100 mm sodium citrate buffer, pH 5 at 45 °C for different time periods (0–24 h). The remaining β‐glucosidase activity was measured as described above. The experiment was conducted in triplicate.

### Determination of kinetic parameters

The Michaelis–Menten kinetics parameters (*V*_max_ and *K*_m_) of hydrolysis of 4‐MUG and *p‐*NPG by the purified enzyme were determined by prisma 7 software using nonlinear regression with different substrate concentrations of the substrates (0–15 μm and 0–17.5 mm for 4‐MUG and *p‐*NPG, respectively) in 100 mm sodium citrate buffer, pH 5 at 45 °C.

### Transglycosylase activity

A reaction mixture consisting of 48 μg of the purified enzyme, 0.02% sodium azide, 100 mm citrate buffer, pH 5.0, and 2.5 μg of cello‐oligosaccharides of different length (cellobiose, cellotriose, and cellotetraose, Seikagaku America, Associates of Cape Cod, Inc., MA, USA), in a final volume of 400 μL, were incubated at 45 °C. After 10 h of incubation, 1 mL of cold acetone was added and the samples were incubated at −20 °C for 30 min. Samples were centrifuged at 10 000 ***g*** for 10 min and the acetone from the recovered supernatant was evaporated. The samples were freeze dried and resuspended in the mínimum volume.

Hydrolytic products from cello‐oligosaccharides were analyzed by TLC using a silica gel TLC plate (Silica gel 60 F254; Merck Millipore, Darmstadt, Alemania) in a solvent system of butanol : 2‐propanol : water (3 : 12 : 4, by vol.) and the released sugars were visualized using a silver stain 25. Standard sugars: glucose (G1) at 1 μg, cellobiose (G2) at 2 μg, cellotriose (G3) at 4 μg, and cellotetraose (G4) at 4 μg.

### SDS/PAGE

Purified protein pattern was analyzed through a 7% SDS/PAGE. The protein was stained with Flamingo^™^ fluorescent stain (Bio Rad Mexico, CDMX, Mexico). Relative molecular mass (*M*_r_) was calculated by comparison with the migration of the standard proteins (Bio‐Rad, SDS/PAGE Broad Range, 161–0317).

### Zymogram analysis

For detection of β‐glucosidase activity through zymogram techniques a 7%, SDS/PAGE or native PAGE, were performed at 4 °C. In both conditions, the sample was incubated for 30 min at 37 °C in sample buffer. At the end of the electrophoresis, gels were rinsed 2× in water and 3× in 100 mm sodium citrate buffer, pH 5, for 5 min each. This was followed by incubation for 1 h at 45 °C in the presence of 4‐MUG (5 μm) as substrate. The zymogram gel was visualized under UV light to detect fluorescence due to the 4‐MU released.

## Results

### Growth kinetics and secreted β‐glucosidase activity

**Figure 1:**
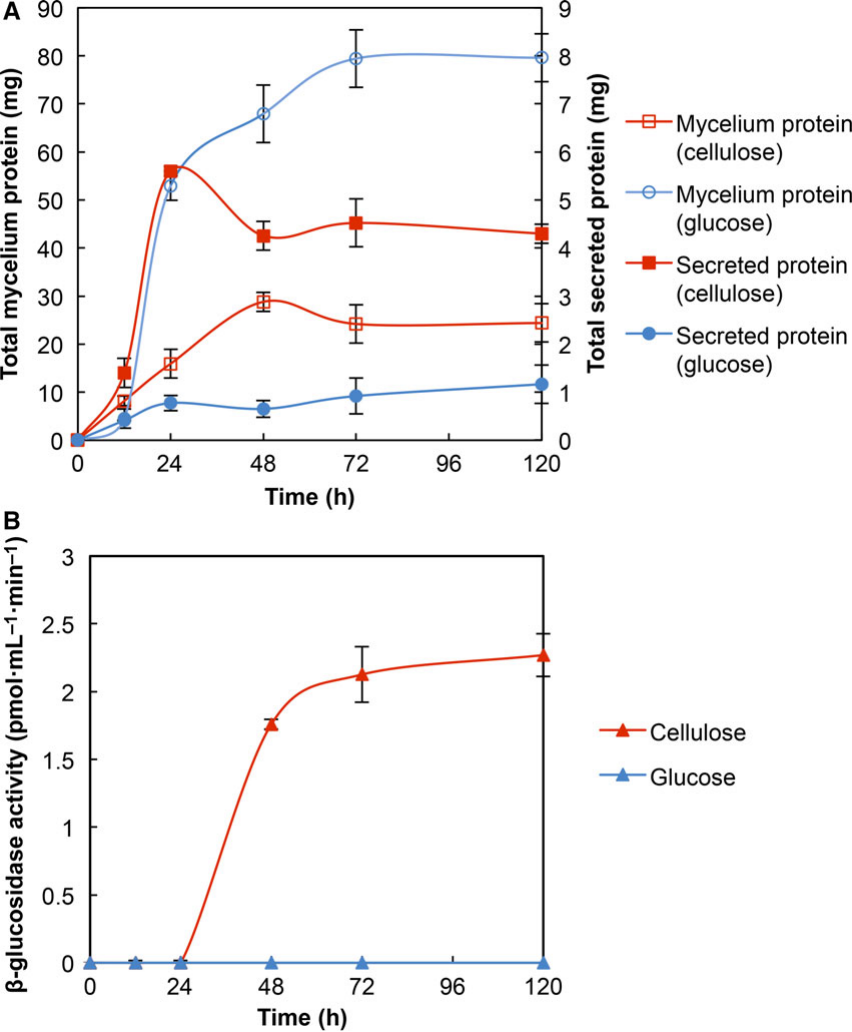
Growth kinetics of the mycelium form of *Sporothrix schenckii* and secreted β‐glucosidase activity. (A) Growth and secretion of protein by *S. schenckii* in the presence of 2% glucose (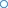, mycelium protein; 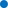, secreted protein) or under induction conditions with 2% cellulose (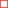, mycelium protein; 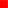, secreted protein). (B) Secreted β‐glucosidase activity in the presence of 2% glucose (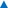) or 2% cellulose (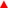).

*Sporothrix schenckii* showed a higher growth rate when the medium was supplemented with glucose than cellulose (Fig. 1A), possibly due to cellulose crystalline nature that makes its degradation more complex, diminishing the fungus growth. The amount of extracellular protein when the fungus was grown in the presence of cellulose was four times higher than when glucose was added as the sole carbon source, suggesting the secretion of different enzymes in order to degrade the polysaccharide.

The extracellular β‐glucosidase activity was determined by using 4‐MUG as substrate and measured as described in Experimental procedures. It was detected after 24 h of incubation and showed the highest activity at 72 h remaining constant through 120 h (Fig. 1B). This behavior suggests the action of previous enzymes such as endoglucanases and cellobiohydrolases acting on the cellulose to generate oligosaccharides which can be subsequently hydrolyzed by β‐glucosidase.

### Purification of *S. schenckii* β‐glucosidase

**Figure 2:**
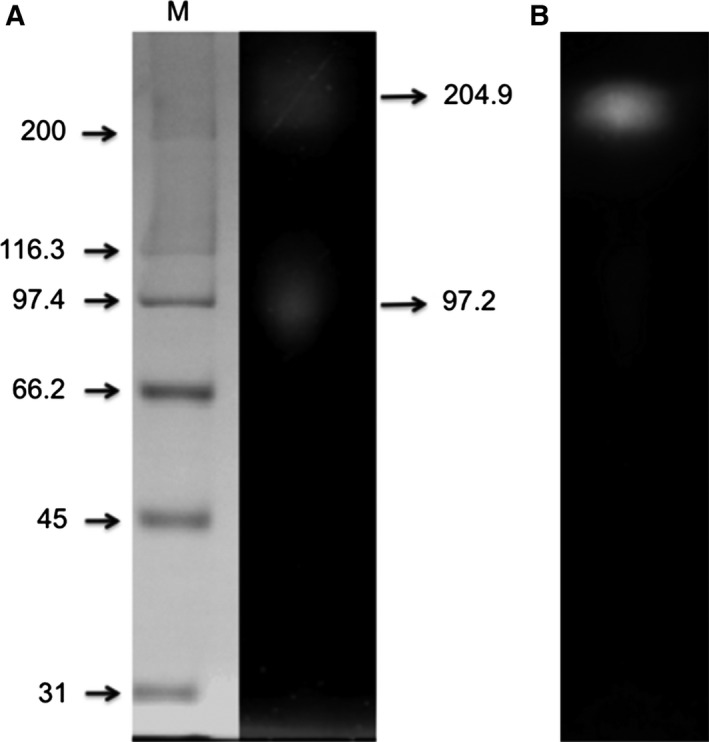
Zymogram of the β‐glucosidase activities from the CFCF (cell‐free concentrated filtered) recovered after 3 days of incubation. (A) Denaturated‐zymogram and (B) native‐zymogram. M, molecular weight standards.

Previous to the purification, zymogram gels were conducted to confirm the presence of β‐glucosidases and to estimate their molecular weight when the fungus was incubated in cellulose as the sole carbon source. Under semidenaturated conditions, we observed two bands with molecular weights of 204.9 and 97.2 kDa (Fig. 2A) while in native‐zymogram we obtained only the high molecular weight band (Fig. 2B).

**Figure 3:**
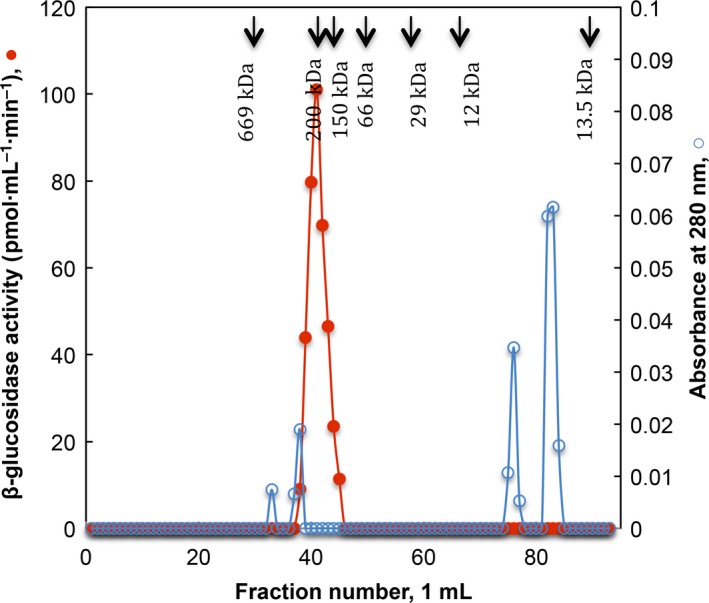
Sephacryl S‐200 HR gel filtration of β‐glucosidase activity. Profile elution of β‐glucosidase activity (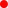) and protein (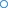). Elution position of standard proteins is indicated with the arrows.

**Figure 4:**
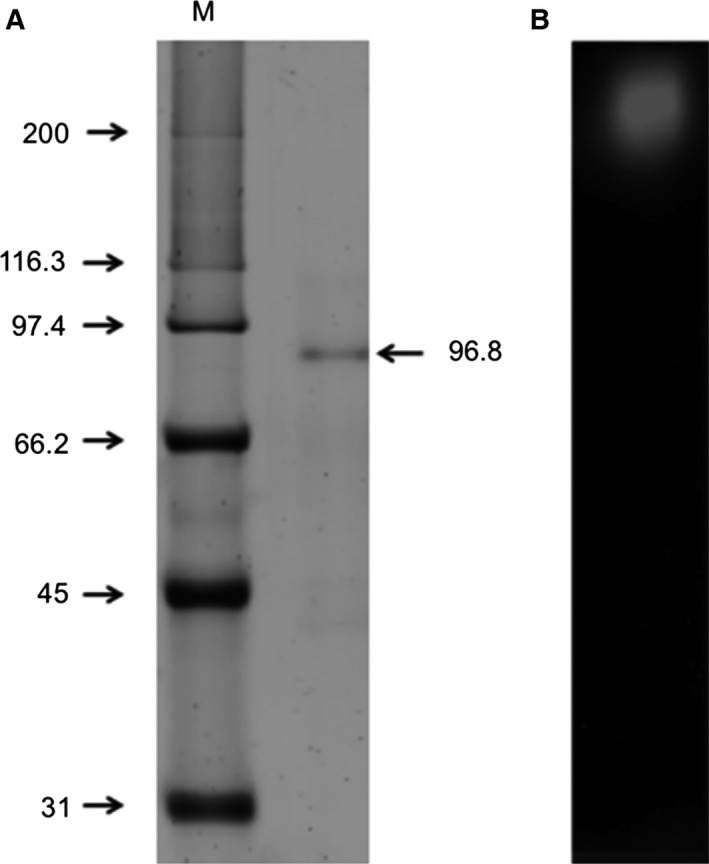
SDS/PAGE and zymogram of the purified β‐glucosidase. (A) Purified enzyme stained with Flamingo (Bio‐Rad) and (B) native‐zymogram. M, molecular weight standards.

The enzyme was purified to homogeneity in a two‐step protocol. Previous to its adsorption in hydroxyapatite resin (HAp), the sample was treated as described in methods. The enzymatic activity in CFCF did not interact with the HAp resin and it was recovered in the initial eluted fractions. Contaminant proteins were removed with 10 mm and 1 m phosphate buffer, pH 7. Fractions with β‐glucosidase activity were concentrated and applied to Sephacryl S200‐HR size exclusion chromatography. β‐glucosidase activity was recovered in a single symmetric enzymatic activity peak (fractions 39–43) with an estimated *M*_r_ of 197 kDa and undetectable protein measured at A_280_ (Fig. 3). Fractions with positive activity were pooled and concentrated to determine its purity and to performe its biochemical characterization. Figure 4 shows the SDS/PAGE analysis (Fig. 4A) of the purified enzyme where only one protein band is exhibited with a calculated *M*_r_ of 96.8 kDa and a zymogram under native conditions (Fig. 4B). A summary of the purification results is given in Table 1. Due to culture interferences to determine protein, the calculation of purification, was made after the remotion of salts. The enzyme was purified to homogeneity with a yield of 50.2% and a specific activity of 800.80 U·mg·protein^−1^.

**Table 1:** Summary of the purification of *Sporothrix schenckii* β‐glucosidase. 1 unit (U) = 1 nmol methylumbelliferone released from methylumbelliferyl‐β‐D‐glucoside per min

Purification steps	Total protein (mg)	Total activity (U)	Specific activity (U·mg·protein^−1^)	Fold purification	Yield (%)
Bio‐Gel P‐10	6.15	1.531	0.249	1.0	100.00
HAp (in batch)	4.40	1.225	0.278	1.1	80.01
Sephacryl S200‐HR	0.96	0.769	0.801	3.2	50.20

### Biochemical characterization of β‐glucosidase

**Figure 5:**
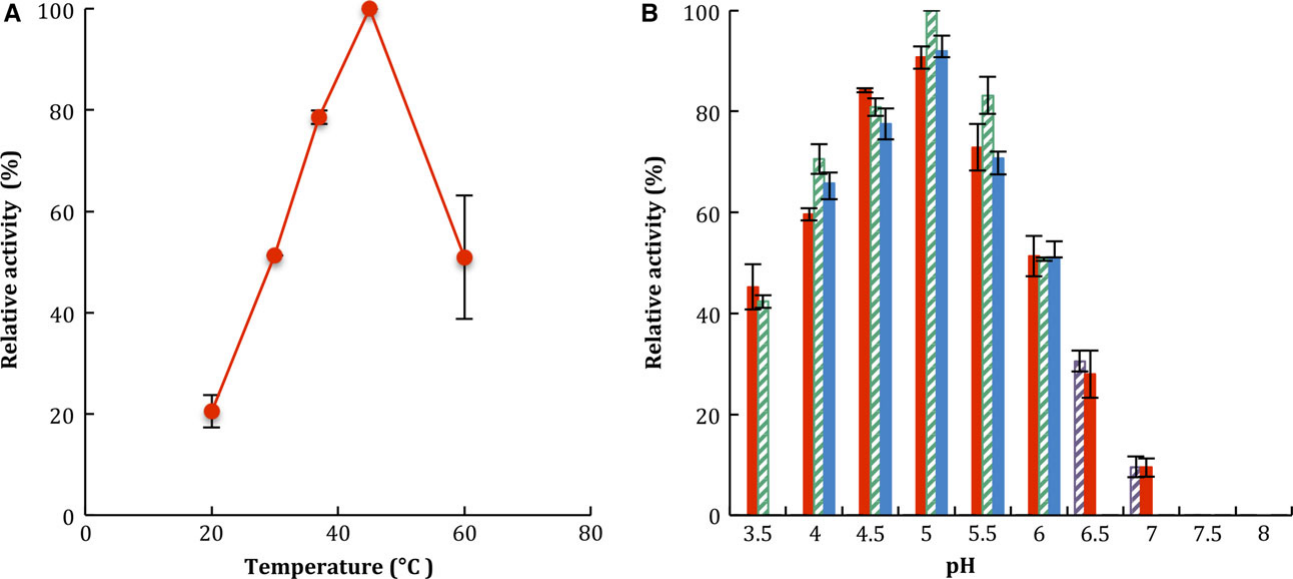
Effects of temperature (A) and pH (B) on β‐glucosidase activity from *Sporothrix schenckii*. To determine the effect of temperature on the activity, purified enzyme was incubated with 4‐MUG as substrate at 20–60 °C in 100 mm citrate buffer pH 5. To determine the effect of pH on the activity, purified enzyme was incubated with 4‐MUG as substrate in 100 mm following buffers: phosphate, pH 6.5–8.0 (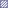), citrate‐phosphate, pH 3.5–8.0 (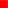), citrate, pH 3.5–6.0 (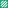), acetate, pH 4.0–6.0 (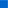). Data are expressed as relative activity (%) where the maximum specific activity is 0.801 nmol of 4‐MU·mg^−1^·min^−1^. Values are means ± SD for three independent experiments.

**Figure 6:**
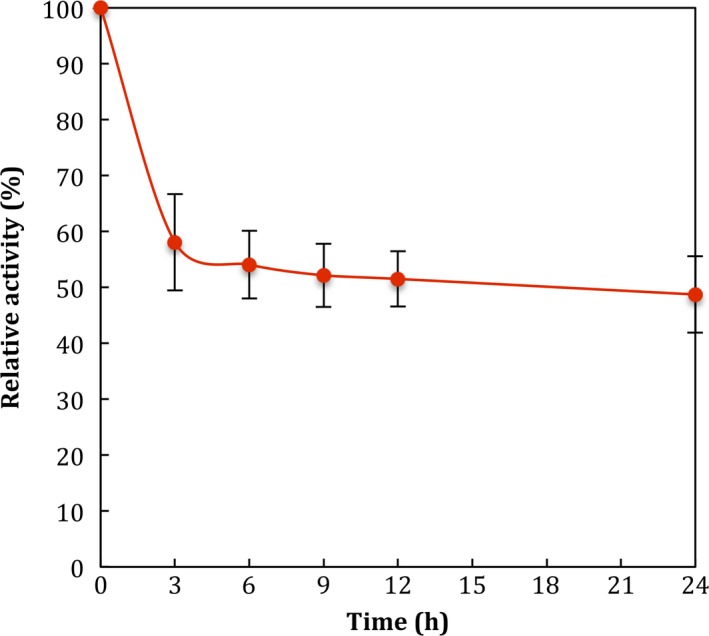
Effect of temperature on the stability of purified β‐glucosidase from *Sporothrix schenckii*.

**Figure 7:**
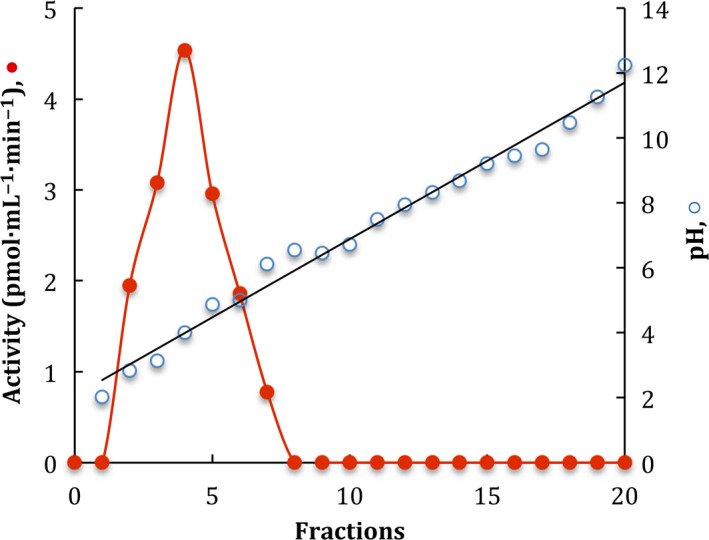
Isoelectrofocusing of the purified β‐glucosidase. Twenty fractions were collected and pH and β‐glucosidase activity (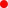) were determined.

The β‐glucosidase activity was measured at different temperatures and pH values (Fig. 5). The optimum temperature determined for the purified enzyme was 45 °C (data not shown) and it was relatively stable when incubated at 45 °C for 24 h, retaining 60% of its initial activity (Fig. 6). Maximum enzyme activity was at pH 4.5–5.5 with citrate, acetate, or citrate‐phosphate buffers and no activity was observed at pH values above 7.5 using TRIS‐HCl and phosphate buffers (Fig. 5B). The pI value of the purified enzyme was determined with a preparative electrofocusing using a Rotofor (Bio‐Rad). The results showed a pI value in a range of 3.2–4.9 with the highest value at 4.0 (Fig. 7).

The effect of divalent ions and chelating agents were tested on the purified β‐glucosidase at different concentrations: 2.5, 5.0, and 10.0 mm (Table 2). The β‐glucosidase activity was only inhibited by Fe^2+^ addition and was not significantly influenced by any tested reagents.

**Table 2:** Effect of divalent ions and chelating agents on β‐glucosidase activity. Values are means ± SD for three different experiments

Metal ion	Relative activity (%)^a^		
	2.5 mm	5.0 mm	10.0 mm
None	100 ± 0.2	100 ± 0.2	100 ± 0.2
Ca^2+^	99 ± 1.9	99 ± 1.6	97 ± 1.1
Co^2+^	99 ± 2.9	96 ± 4.9	94 ± 1.8
Cu^2+^	93 ± 4.0	96 ± 5.5	93 ± 5.8
Fe^2+^	72 ± 1.6	40 ± 1.3	19 ± 2.4
Mg^2+^	95 ± 5.1	94 ± 0.6	96 ± 1.3
Mn^2+^	98 ± 1.8	97 ± 2.1	95 ± 4.2
EDTA	98 ± 2.2	98 ± 1.5	96 ± 3.0
EGTA	95 ± 3.5	97 ± 4.5	97 ± 3.9

a 100% activity corresponds to 0.801 nmol·min^−1^·mg^−1^ protein.

According to nonlinear regression, Michaelis–Menten enzyme kinetics calculated were, the *K*_m_ value for *p*‐NPG (44.14 mm ± 6.11) was higher than *K*_m_ for 4‐MUG (0.012 mm ± 0.001), whereas the *V*_max_ values were 22.49 ± 0.28 and 2.56 ± 0.31 nmol·mg^−1^·min^−1^ for *p‐*NPG and 4‐MUG, respectively (data not shown).

Substrate specificity was assayed using 4‐MU‐glycosides, *p*‐NP‐glycosides, and saccharides as substrates under the standard assay conditions (Table 3). Purified β‐glucosidase showed 2.2× higher specific activity on cellobioside than laminarin and 7.2× higher specific activity on *p‐*NPG than 4‐MUG. It also evidenced low activity on the cellobiose derivates, 4‐MUC, and *p‐*NPC. No activity was detected on 4‐MU‐β‐D‐xyloside or 4*‐*MU‐β‐D‐galactopyranoside.

**Table 3:** Substrate specificity of the purified enzyme

Substrate	Specific activity (nmol·mg^−1^·min^−1^)
Synthetic substrates	
4MU‐β‐D‐glucoside	0.75 ± 0.010
4MU‐β‐D‐cellobioside	0.04 ± 0.006
4MU‐β‐D‐xyloside	N.D.
4MU‐β‐D‐galactoside	N.D.
*p*‐Nitrophenyl β‐D‐glucopyranoside	5.40 ± 0.038
*p*‐Nitrophenyl β‐D‐cellobioside	0.53 ± 0.081
Saccharides	
Cellobiose	107.41 ± 14.87
Laminarin	59.78 ± 9.40

N.D., not detected. Depending on the substrate, activities were determined by measuring the release of either 4‐MU, *p*‐NP, or glucose as described in Experimental procedures. The concentration of synthetic substrates was 5 μm (4‐MU substrate) and 5 mm (*p*‐NP substrates), whereas the concentration of saccharides was 1% (w/v). The specific activity is expressed as nmol of substrate hydrolyzed per minute per mg of protein. Values are means ± SD for three independent experiments.

**Figure 8:**
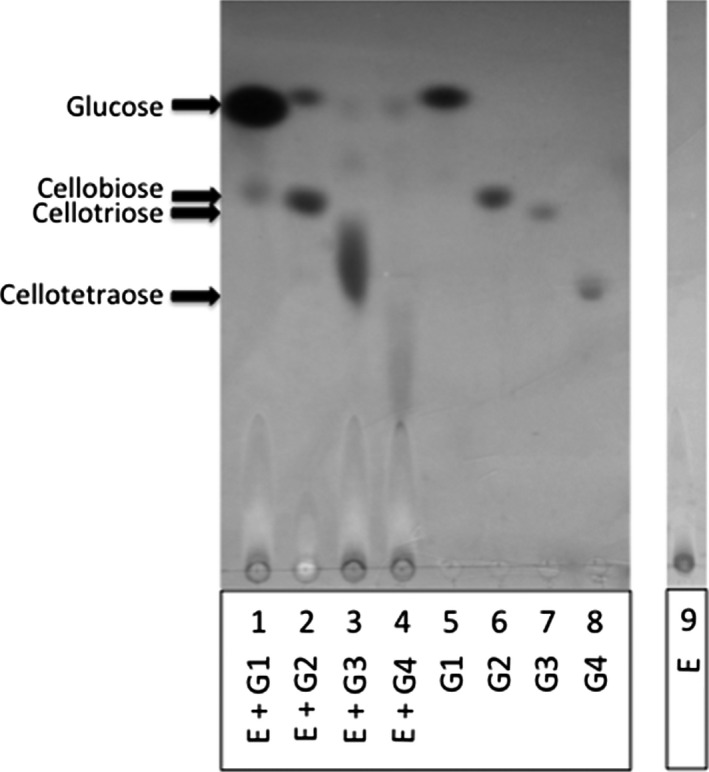
Thin‐layer chromatography separation of the released cellodextrins when purified enzyme was incubated with glucose (E + G1), cellobiose (E + G2), cellotriose (E + G3) or cellotetraose (E + G4). Reaction mixtures were stopped after 10 h. Glucose [G1 (1 μg), line 5], cellobiose [G2 (2 μg), line 6], cellotriose [G3 (4 μg), line 7] and cellotetraose [G4 (4 μg), line 8] were used as standards and purified β‐glucosidase as control (E, line 9).

In order to evaluate the hydrolysis on cellodextrins by the purified β‐glucosidase, we performed a TLC (Fig. 8). The enzyme was able to release glucose from cellobiose > cellotetraose > cellotriose. Apparently, glucose was the unique product from the hydrolysis of cellobiose (Fig. 8, line 2). When cellotriose (Fig. 8, line 3) was used as substrate, a strong signal of a probable tetrasaccharide and two diffuse spots, corresponding to glucose and a possible disaccharide, were showed. Using cellotetraose (Fig. 8, line 4) as substrate, only two spots appeared, one comparable to glucose mobility and other with a relative mobility lower than a tetrasaccharide. Additionally, when the enzyme was incubated in the presence of glucose (Fig. 8, line1), a glucose dimer appeared, suggesting transglycosylation activity. A similar effect was observed when cellotriose and cellotetraose were used in the assay (Fig. 8, line 3 and 4), spots with lower mobility were visualized, indicating the formation of a bigger oligosaccharide.

## Discussion

Despite being a human pathogenic fungus, *S. schenckii* has a saprophytic stage and it is phylogenetically close to another phytopathogenic and nonpathogenic fungus which secretes different hydrolytic enzymes 7, 8, 9. Recently, the characterization of 36 different isolates of *S. schenckii* complex based on the hydrolysis of 11 substrates 26 demonstrated that *S. schenckii* possesses a battery of extracellular lytic enzymes which may help it to obtain necessary nutrients for its development and maintenance during its saprophytic phase.

In the present work, we confirmed that *S. schenckii* produces and secretes enzymes capable of degrading cellulose, one of the main cell wall component of organic matter in which it develops. This fungus, in the presence of cellulose as the principal carbon source, produced considerable amounts of β‐glucosidase. It is known that this enzyme acts mainly on cellobiose and it is needed for an efficient degradation of cellulosic biomass as it reduces the inhibition of cellobiohydrolases by decreasing the product accumulation. β‐glucosidase has been well characterized in another fungus: opportunistic such as *Aspergillus* spp. 27, 28, phytopathogenic such as *Fusarium oxysporum*, 29 and saprophytic such as *Neurospora crassa* 8, 30, 31.

Under the tested growth conditions, *S. schenckii* secreted low quantity of protein. As part of this secreted proteins, we detected β‐glucosidase activity. The induction pattern of β‐glucosidase activity showed an increase in early stationary phase (72 h) and remains constant throughout the tested time (120 h). Similar results were shown in *Aspergillus niger* 32. This result is in contrast with other cellulases reported in other fungi, where the highest cellulolytic activity is expressed after 9–10 days of growth 33, 34, 35. When *S. schenckii* was grown in glucose, lower amount of protein was secreted and β‐glucosidase activity was not detected, however, it does not dismiss the possibility that low levels of constitutive enzyme could be present and cellulase induction could require its own basal expression as it has been previously reported in *T. reesei* 36. In addition of the secreted hydrolytic enzymes, the fungus produced a metabolic product that turned the culture medium yellow when it was grown in the presence of cellulose. Since this dye interfered with protein determination, the CFCF was eluted in Bio‐Gel P‐10 in order to remove it. After this filtration step, another two chromatographies were performed to purify to homogeneity a β‐glucosidase from *S. schenckii* with a 50% yield and 3.2‐fold of purification. Purified fungal β‐glucosidases present a wide diversity regarding the yield and the fold purification. While some reports had described the purification to homogeneity of fungal β‐glucosidases with high yields 11, 29 apparently, an overall characteristic of this enzymes is the low recovered rate 37, 38, 39 and the low fold purification 40, 41, suggesting its probable lability. In *Penicillium italicum* and *Penicillium simplicissimum*, an enzymatic activity recovery rate and fold purification has been reported similar to those obtained in the present study 42, 43.

The enzyme purified after filtration on Sephacryl S200‐HR was applied to an SDS/PAGE and the electrophoretic profile showed a single band with an estimated molecular weight of 96.8 kDa. The native molecular weight of the enzyme (197 kDa) determined by gel filtration on size exclusion chromatography suggests that the purified enzyme has a dimeric conformation, as observed in *N. crassa* intracellular β‐glucosidase 44. We were not able to obtain the denatured zymogram of the purified enzyme (data not shown), probably due to its lability as an unprotected single protein. Nevertheless, when the CFCF zymogram was performed under denatured conditions, two bands were exhibited (204.9 and 97.2 kDa), one of them with a similar molecular weight to the one displayed in the native zymogram of the purified protein. This result confirms the secretion of a dimeric protein with β‐glucosidase activity induced by cellulose. In other β‐glucosidases from other microorganisms, it was was demonstrated that the oligomeric conformation is not necessary for enzymatic activity 45. In contrast to these results 46, a barley β‐D‐glucan exohydrolase with β‐D‐glucosidase activity was reported and it was suggested that space at the interface between the two domains of the homodimeric protein is probably the active site of the enzyme.

Purified β‐glucosidases isolated from different microorganisms present a variability with respect to their physicochemical properties. Reported fungal β‐glucosidases display optimal pH values between 3 and 6 and are active in a broad range of temperatures from 40 to 60 °C 47. *Sporothrix schenckii‐*purified β‐glucosidase exhibit an optimal temperature and pH of 45 °C and 5.0, respectively. Similar values have been reported from other ascomycetes, such as *N. crassa* 48 and *Xylaria regalis* 37. Divalent cations, such as Ca^2+^, Mg^2+^, Co^2+^, and Mn^2+^ can affect the enzyme activity of β‐glucosidases by activating 37, 41 or inhibiting them 49. We only observed an inhibitory effect in the presence of 10 mm Fe^2+^ and no other effect with the tested reagents. Similar results were reported from purified β‐glucosidase from the wood‐decaying fungus *Daldinia eschscholzii* (Ehrenb.:Fr.) Rehm 39.

Interestingly when the thermostability of the enzyme was determined, the enzyme retained 60–50% of its initial activity after 3 h of incubation at 45 °C and it was maintained until 24 h of incubation. Although there is a considerable decrease in the enzyme activity during the first 3 h, we suppose that after initial incubation time, the conformation of the protein is no longer affected during the tested times. β‐glucosidases studies carried out in *Melanocarpus* sp. 40 and *A. niger* 11 also showed a significant decrease in β‐glucosidase activity when enzymes were incubated for short periods of time (60 min) at different temperatures ranging from 40 to 70 °C.

The *K*_m_ and *V*_max_ values from *S. schenckii* β‐glucosidase revealed that the enzyme had a 3678‐fold higher affinity to 4‐MUG than *p‐*NPG and can hydrolyze 8.8 times faster the former than the latter. The highest hydrolytic activity of the isolated β‐glucosidase was observed against glucose derivatives (4‐MU and *p*‐NP derivatives) as substrates, detecting only low activity against cellobiose derivatives. Activity was also detected when natural substrates such as cellobiose and laminarin were tested. This suggests the possibility that the purified enzyme could hydrolyze short‐chain cello‐oligosaccharides and other types of linkage (β‐1,3) as previously shown in other β‐glucosidases isolated from different microorganisms 29, 50, 51. This assumption was confirmed by TLC experiments, where the hydrolysis of cello‐oligosaccharides of different length by the purified β‐glucosidase was proved. Beside the detected oligosaccharides hydrolysis, new spots corresponding to higher molecular weight oligosaccharides appeared, suggesting transglycosylation activity in addition to the hydrolytic activity. Results from substrate specificity analysis confirmed the nature of this enzyme as a β‐glucosidase and proved that it can also catalyze both hydrolysis and transglycosylation reactions as reported in *A. fumigatus, A. niger, A. oryzae, Magnaporthe grisea, N. crassa*, and *Penicillium brasilianum* 52, 53.

In order to determine if this enzyme is present in another strain of the *S. schenckii* complex, *Sporothrix brasiliensis* was grown under similar conditions and β‐glucosidase activity was determined using 4‐MUG as substrate. Specific activity was four times lower than the one secreted by *S. schenckii* (data not shown). These data are consistent with a previous report which demonstrates the presence of a secreted β‐glucosidase activity in *S. schenckii, S. brasiliensis,* and *S. albicans* strains using the semiquantitative API‐ZYM commercial kit system (BioMérieux, Marcy‐l’Étoile, France). In contrast, this activity was not found when it was measured in *S. mexicana* and *S. globosa* supernatants using the same methodology 26.

To our knowledge, this work represents the first report of the purification of a β‐glucosidase secreted by the human pathogen fungus *S. schenckii* growth in a complex polysaccharide. The reported purified protein and other secreted enzymes, which have not been identified yet, could provide the ability to *S. schenckii* to grow as a saprophytic fungus. Detailed analysis such as molecular cloning and expression data are needed in order to give us better insights into the saprophytic phase of this fungus.

## Author contributions

AHG, AFM, PPN, and JCVC planned the experiments; AHG, AFM, and JCVC performed the experiments; AHG, AFM, PPN, and JCVC analyzed the data; AHG and JCVC wrote the manuscript.
